# Does behavioural inhibition system dysfunction contribute to Attention Deficit Hyperactivity Disorder?

**DOI:** 10.1017/pen.2019.5

**Published:** 2019-08-08

**Authors:** S. Sadeghi, J. McIntosh, S. M. Shadli, D. Healey, R. Rostami, P. Trani, N. McNaughton

**Affiliations:** 1 Department Psychology, University of Otago, POB 56, Dunedin, New Zealand; 2 Department Paediatrics, University of Otago, POB 56, Dunedin, New Zealand; 3 University of Tehran, 16th Azar Street, Enghelab Square, Tehran, Iran

**Keywords:** ADHD, EEG, inhibition, Attention Deficit Hyperactivity Disorder, behavioural inhibition system, stop signal task

## Abstract

The Reinforcement Sensitivity Theory of Personality has as its main foundation a Behavioural Inhibition System (BIS), defined by anxiolytic drugs, in which high trait sensitivity should lead to internalising, anxiety, disorders. Conversely, it has been suggested that low BIS sensitivity would be a characteristic of externalising disorders. BIS output should lead to increased arousal and attention as well as behavioural inhibition. Here, therefore, we tested whether an externalising disorder, Attention Deficit Hyperactivity Disorder (ADHD), involves low BIS sensitivity. Goal-Conflict-Specific Rhythmicity (GCSR) in an auditory Stop Signal Task is a right frontal EEG biomarker of BIS function. We assessed children diagnosed with ADHD-I (inattentive) or ADHD-C (combined) and healthy control groups for GCSR in: a) an initial smaller study in Dunedin, New Zealand (population ~120,000: 15 control, 10 ADHD-I, 10 ADHD-C); and b) a main larger one in Tehran, Iran (population ~9 [city]-16 [metropolis] million: 27 control, 18 ADHD-I, 21 ADHD-C). GCSR was clear in controls (particularly at 6–7 Hz) and in ADHD-C (particularly at 8–9 Hz) but was reduced in ADHD-I. Reduced attention and arousal in ADHD-I could be due, in part, to BIS dysfunction. However, hyperactivity and impulsivity in ADHD-C are unlikely to reflect reduced BIS activity. Increased GCSR frequency in ADHD-C may be due to increased input to the BIS. BIS dysfunction may contribute to some aspects of ADHD (and potentially other externalising disorders) and to some differences between the ADHD subtypes but other prefrontal systems (and, e.g. dopamine) are also important.

How far is extreme (high or low) sensitivity of the Behavioural Inhibition System (BIS; Gray, 1972, 1982; Gray & McNaughton, 2000) maladaptive? We would expect both high and low trait BIS to be problematic if the system has evolved in the context of balancing selection (Penke, Denissen, & Miller, 2007); we would expect neither (and a narrow range of trait sensitivity) if there is a single optimum. More specifically, if high BIS sensitivity is linked to internalising disorder (as seems to be the case), is low BIS sensitivity linked to externalising disorder? Here, we use a recently developed BIS biomarker (McNaughton, 2018) to begin to address these questions.

There is a good reason to see high sensitivity of the BIS as linked to internalising, particularly anxious, disorder. The BIS (an explicitly neural system centred on the hippocampal formation) is primarily defined by anxiolytic drugs (Gray, 1977; Gray & McNaughton, 2000). Although some may see it as a putative neural system, the functions of which are defined theoretically, both its inputs and outputs were originally identified by their sensitivity to anxiolytics, as was its neurology (Gray, 1982). It was the conjoining of these inputs and outputs that led to postulation of an underlying, anxiolytic-sensitive, system. While the most recent version of the theory now includes an explicit cognitive superstructure to account for the inputs and outputs (Figure 1C), anxiolytic action remains the key defining feature of the BIS and the primary means of determining its neural and cognitive nature (Gray & McNaughton, 2000). In addition to this link with therapeutic drugs, in the guise of trait anxiety, the sensitivity of this neural system has been suggested to relate to neuroticism (Gray, 1970), a trait construct based on the study of those with anxiety disorders (Eysenck & Eysenck, 1964), and that represents a risk factor for them (Andrews, Stewart, Morris-Yates, Holt, & Henderson, 1990).

**Figure 1.**
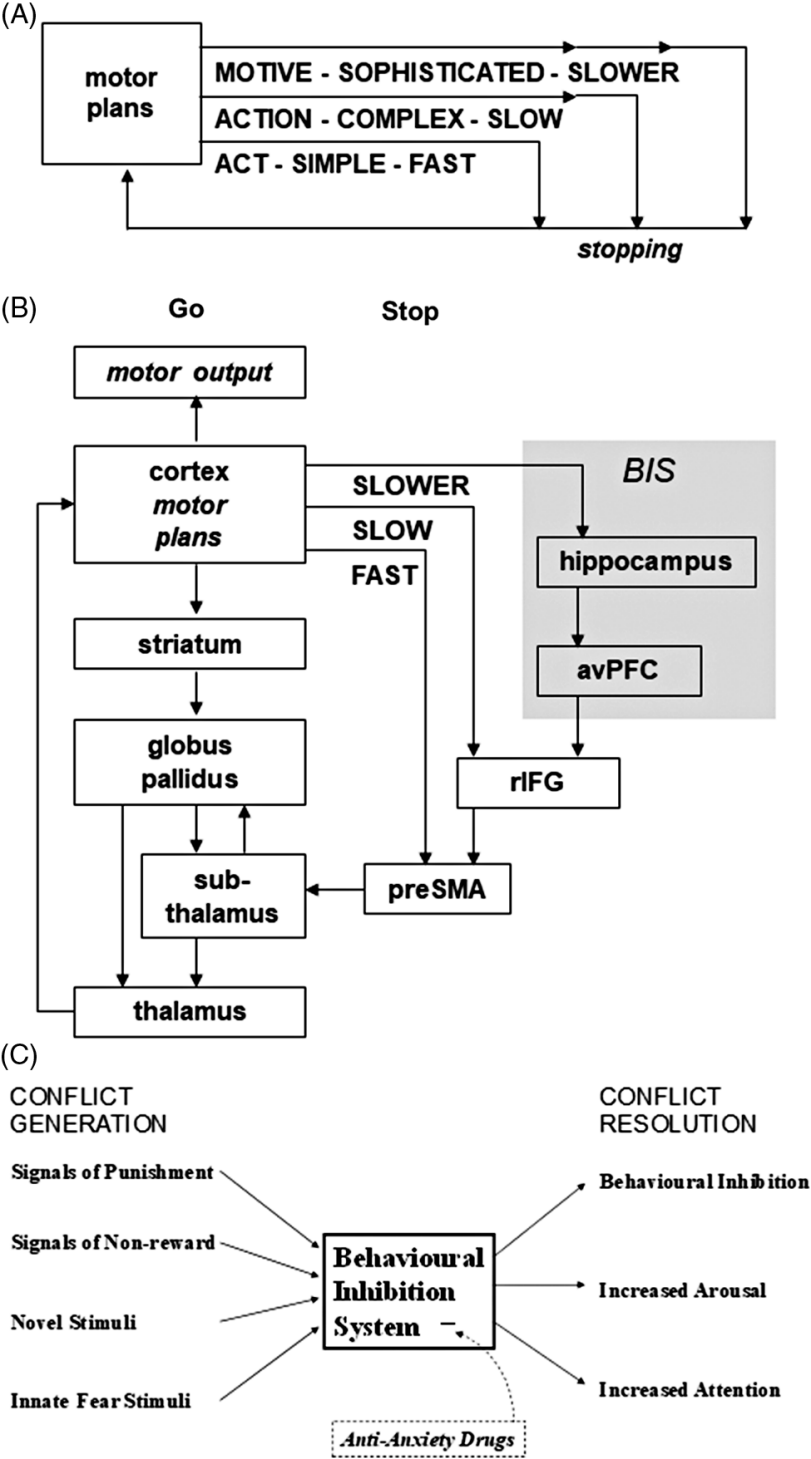
(A and B) Postulated neural control of going and stopping. Motor inhibition uses both fast and slow routes to modulate the go circuit. Goal inhibition involves, in addition, the slower BIS circuit. avPFC = anteroventral prefrontal cortex; rIFG = right inferior frontal gyrus; preSMA = presupplementary motor area. Connections have been simplified, and circuits and structures in the BIS, other than the hippocampus, are not shown (e.g. the Papez circuit is omitted). Figure panels A and B and legend from Neo et al. (2011) with permission. (C) The behavioural inhibition system (Gray, 1982). This responds to any of its adequate inputs (CONFLICT GENERATION) with all of its outputs (CONFLICT RESOLUTION). It comprises the hypothetical substrate on which the anti-anxiety drugs act to reduce anxiety. Note that the key feature of all stimuli which activate the behavioural inhibition system is that they should generate conflict between competing goals. Thus, where a to-be-punished response is weak or where a novel stimulus elicits only approach tendencies uncontaminated with avoidance, the behavioural inhibition system will not be engaged.

*Source*: Figure and legend from Gray & McNaughton (2000) with permission.

The BIS is the most distinctive system (McNaughton & Corr, 2008) within the Reinforcement Sensitivity Theory (RST) of Personality (Corr, 2008). In addition to the BIS, RST assumes important sensitivities for approach and withdrawal systems (as do Cloninger, Svrakic, & Przybecky, 1993; Elliot & Thrash, 2002, 2010). Importantly, trait measures putatively linked to extreme sensitivities of all three motivational systems have been linked to mental disorders (Gomez, Woodworth, Waugh, & Corr, 2012; Mullins-Sweatt & Lengel, 2012; Strelau & Zawadzki, 2011; Trull, 2012). So, a range of psychiatric disorders, unlike neurological or general medical disorders, can be viewed as extremes of variation in the population as a whole of basic biological systems concerned with approach, withdrawal and conflict.

RST, then, sees a range of psychiatric disorders as reflecting extremes of adaptive spectra rather than categorical forms of pathology. The associated traits may be (for both internalising and externalising) neurobiological vulnerabilities that interact in complex ways with “environmental adversities” (Beauchaine, Zisner, & Hayden, 2018); and it may be “that the major dimensions of psychopathology correspond to major trait dimensions of personality, but … extremity on these dimensions is neither necessary nor sufficient for psychopathology, which requires cybernetic [i.e. goal control] dysfunction” (DeYoung & Krueger, 2018, abstract). Nonetheless, if we look at diagnosed cases, we would expect extreme trait sensitivities (even if not vice versa). BIS dysfunction may have some involvement in all externalising disorders (Corr & McNaughton, 2016); but its three distinct outputs most clearly overlap symptoms of Attention Deficit Hyperactivity Disorder (ADHD), so this is one example where we might expect to find extreme low BIS sensitivity.

ADHD is one of the most common childhood disorders (Polanczyk, Silva de Lima, Horta, Biederman, & Rohde, 2007); but its neurology is poorly understood. Its key features are hyperactivity, inattention, and impulsivity that occur in varying combinations – currently classified into three main subtypes (hyperactive/impulsive; inattentive; combined). The neurology of ADHD, and whether this can define a subtype, is unclear (Cortese et al., 2012; Sharma & Couture, 2014; Xia, Foxe, Sroubek, Branch, & Li, 2014). Indeed, rather than being distinct, the subtypes may represent positions within an ADHD (Baeyens, Roeyers, & Walle, 2006; Lemiere et al., 2010; Miller, Derefinko, Lynam, Milich, & Fillmore, 2010), or even externalising (Beauchaine & McNulty, 2013; Beauchaine, McNulty, & Hinshaw, 2016; Krueger et al., 2002; Sellbom, 2016) spectrum.

Hyperactivity, inattention, and impulsivity in ADHD may reflect cognitive, particularly, executive dysfunction resulting in high behavioural activation, low behavioural inhibition, or both. Symptoms appear to arise from both attentional problems during response preparation and inhibitory problems during response execution (Albrecht et al., 2013). Improvement in cognitive performance with age in ADHD appears more linked to changes in low than high executive demand processes (Coghill, Hayward, Rhodes, Grimmer, & Matthews, 2014). Superficial higher order deficits may, then, be consequences of dysfunction of lower order behavioural activation or behavioural inhibition in a hierarchical relationship where inhibition deficits could be fundamental (Barkley, 1997b; Barkley, Grodzinsky, & DuPaul, 1992).

Quay (1997) suggested that ADHD symptoms reflect underactivity in the BIS. While defined by the action of anxiolytic drugs, BIS can also be characterised as being activated by goal conflict (Gray & McNaughton, 2000), and as producing outputs to resolve this that include increased arousal and attention as well as behavioural inhibition (Gray, 1982). So, low trait BIS should produce all three of the main types of ADHD symptom: poor attention, reduced arousal, and behavioural disinhibition. Quay’s interpretation of the original BIS theory (Gray, 1982) predicted that children with ADHD would show longer stop signal reaction times (SSRTs) during Stop Signal Tasks (SSTs) (Quay, 1997); and they have demonstrated slower and more variable median reaction times (MRTs) and SSRTs than typically developing children (Nichols & Waschbusch, 2004).

The SST is also the task from which we extracted a human biomarker for BIS sensitivity. We recorded brain activity in the SST and used an analysis based on the role of goal conflict in BIS theory (Gray & McNaughton, 2000) to produce an EEG measure of BIS sensitivity. To do this, we took advantage of the fact that the SST tracks 50% correct stopping. In order to assess SSRT, the SST has varying stop signal delays (SSDs); so going may be easier than stopping, stopping easier than going, or stopping and going matched (at which point there is 50% correct stopping), which maximises their conflict. Balanced go-stop conflict (compared to the average of brain activity over greater going and greater stopping) in the SST was found to specifically elicit 5–12 Hz rhythmicity at right frontal sites. That is, this task, coupled with an appropriate statistical contrast of the imposed conditions, elicited goal-conflict-specific rhythmicity (GCSR). This GCSR correlated with neuroticism and trait anxiety (Neo, Thurlow, & McNaughton, 2011; Shadli, McIntosh, Glue, & McNaughton, 2015). It should be noted that the conflict-specific contrast (intermediate stopping vs. the average of short and long) is quite distinct from previous studies of right frontal evoked response potentials in ADHD with the SST, where the average of all stop-related activity is used (Albrecht, Banaschewski, Brandeis, Heinrich, & Rothenberger, 2005; Dimoska, Johnstone, Barry, & Clarke, 2003; Pliszka, Liotti, & Woldorff, 2000).

The BIS theory has extensive behavioural, cognitive and neural elaboration (Gray & McNaughton, 2000); and it is its cognitive components that were used to develop GCSR (Neo et al., 2011; Shadli et al., 2015). But, as we have already noted, the BIS is defined by anxiolytic drug action (Gray, 1977, 1982); and a key foundation for the theory is that a reduction in the frequency of 5–12 Hz rhythmicity elicited in the hippocampus of freely moving rats (McNaughton & Sedgwick, 1978) has predicted clinical anxiolytic action for 40 years with no false positives or negatives (McNaughton, Kocsis, & Hajos, 2007). It is important, therefore, that GCSR in the SST was sensitive to all three major classes of anxiolytic drugs that do not also have both anti-panic and anti-depressant actions (McNaughton, Swart, Neo, Bates, & Glue, 2013; Shadli et al., 2015).

Importantly, the anxiolytic drugs that define the BIS do not affect SSRT in the SST (McNaughton et al., 2013; Shadli et al., 2015). So, in addition to the separate parallel act (fast) and action (slow) systems previously shown to control SSRT (Aron & Poldrack, 2006; Floden & Stuss, 2006), there appears to be a parallel goal (even slower) inhibition system that is activated in the SST but with too long a delay to affect behaviour (Figure 1).

The fact that stopping in the SST is insensitive to anxiolytics and so is not controlled by the BIS opens the question of whether the BIS contributes to ADHD. Here, we directly tested whether Gray’s BIS theory can explain some of the deficiencies seen in ADHD. We have previously predicted (Corr & McNaughton, 2016) that ADHD groups would show lower BIS responses than neurotypical controls, consistent with Quay’s (1997) suggestion of BIS (goal conflict) involvement in ADHD but distinct from any contribution to ADHD from act/action stopping. We tested for differences between the inattentive and combined types of ADHD but did not include hyperactive/impulsive as these are very rare.

## 1. Methods

Two studies were undertaken, one in New Zealand (initial study) and one in Iran (main study). All procedures were in accordance with the ethical standards of the University of Otago Human Ethics Committee (approval number: 10/043) and with the Helsinki Declaration of 1975, as revised in 2008.

### 1.1 Participants and selection criteria

The initial study had 31 participants in the final analysis (15 control, 7 inattentive, 9 combined; with a further 23 excluded due to excessive, >15%, artefacts in their EEG recordings and lower than 50% of correct responses in stop trials). The main study had 66 participants (27 control, 18 inattentive, 21 combined; with a further 12 excluded as per the initial study). For both, age ranged from 7 to 12 years with an average of 9. In the initial study, participants (including controls) were mostly selected by a research assistant from the Early Learning Database at the University of Otago, Department of Psychology. This database contains people invited at birth to go on a research database to be invited to participate in studies in the Psychology Department of the University of Otago. A diagnostic assessment using the Kiddie Schedule for Affective Disorders and Schizophrenia (K-SADS) semi-structured interview with parents (Kaufman et al., 1997), combined with parent and teacher ratings, was carried out by a registered clinical psychologist using standard DSM-IVR criteria. Some ADHD participants were referred by a paediatrician from the University of Otago, Department of Psychological Medicine. In the main study, participants in the control group were children selected from two primary schools (one for girls and one for boys) in Tehran. The schools were asked to refer their students for a free integrated visual and auditory continuous performance test. If the test results were normal and parents did not report any ADHD-like symptoms, they were invited to take part. If their results were not normal, we recommend their parents to take them to visit a psychologist or psychiatrist for further examination. Participants in the experimental groups were children with ADHD selected from the database of the Atieh Clinic, a childhood psychological disorders clinic in Tehran. The children were first interviewed by a psychiatrist, then they and their parents provided responses on Conners’s rating scale (Conners, Sitarenios, Parker, & Epstein, 1998) and then tested with IVA + plus^TM^ (BrainTrain, Inc, North Chesterfield, VA 23235, USA). A psychiatrist used all three to provide a DSM-IVR diagnosis.

Geographical (including linguistic) and temporal separation of the studies prevent matching of diagnostic criteria and so tests the generalisability of the results obtained. In both studies, we contacted parents by phone and then emailed them an information package. Parents received an NZ$10 dollar (or equivalent) petrol voucher in recognition of the time and costs of attending. Both parents and children received information sheets and then signed informed consent forms before starting the test. We asked children to be off medication for 24 h before testing. We did not check for other conditions, like anxiety, in the control or experimental groups (see section 4.2).

### 1.2 Procedure

Children arrived at the laboratory with a parent for a 40-min session. After signing the consent forms, they were moved into an electrically body-protected room (i.e. with power controlled by a residual current device). They sat on a chair and the researcher accompanied them to ensure that they followed the test instructions. The stimuli were presented on a 14-inch monitor at eye level at a distance of 90–120 cm from the face and the stop signal tone was presented by headphones worn over the ears. All aspects of the experiment were controlled by a program written in Visual Basic 6. In the initial study, all participants were fitted with a Wave Guard cap with Ag/AgCl electrodes and mastoid reference electrodes (A1, A2). A1 and A2 were averaged to create a single reference for processing to which all channels were re-referenced. EEG data were recorded from 19 International 10–20 electrode channels: F7, F3, Fz, F4, F8, T3, C3, Cz, C4, T4, T5, P3, Pz, P4, T6, O1, O2, Fp1 and Fp2. Statistical analysis for the current report was focussed, a priori, on the right frontal channel F8 as previous studies in adults (Neo et al., 2011; Shadli et al., 2015) have found conflict power correlated with neuroticism and trait anxiety most reliably at F8. Electro-gel (Electro Cap International, USA) was inserted with a 3 ml syringe with a Precision Glide 16 gauge blunt needle (Becton & Dickenson & Co, USA) into electrodes to aid conductance. Impedances were checked via the ANT Neurotechnology system and adjusted to below 5 kΩ. In the main study, EEG recording was via an Electrocap (Electro Cap International, USA) with pure tin electrodes connected to a Mitsar-EEG amplifier system with WinEEG software and referenced to two pure tin ear electrodes. Other details were as for the initial study. Sampling rate was 256 Hz (1.6–30 Hz bandpass) in the initial study and 250 Hz (2–36 Hz bandpass) in the main study, with both resampled to 128 Hz for analysis.

### 1.3 Stop signal task

**Figure 2.**
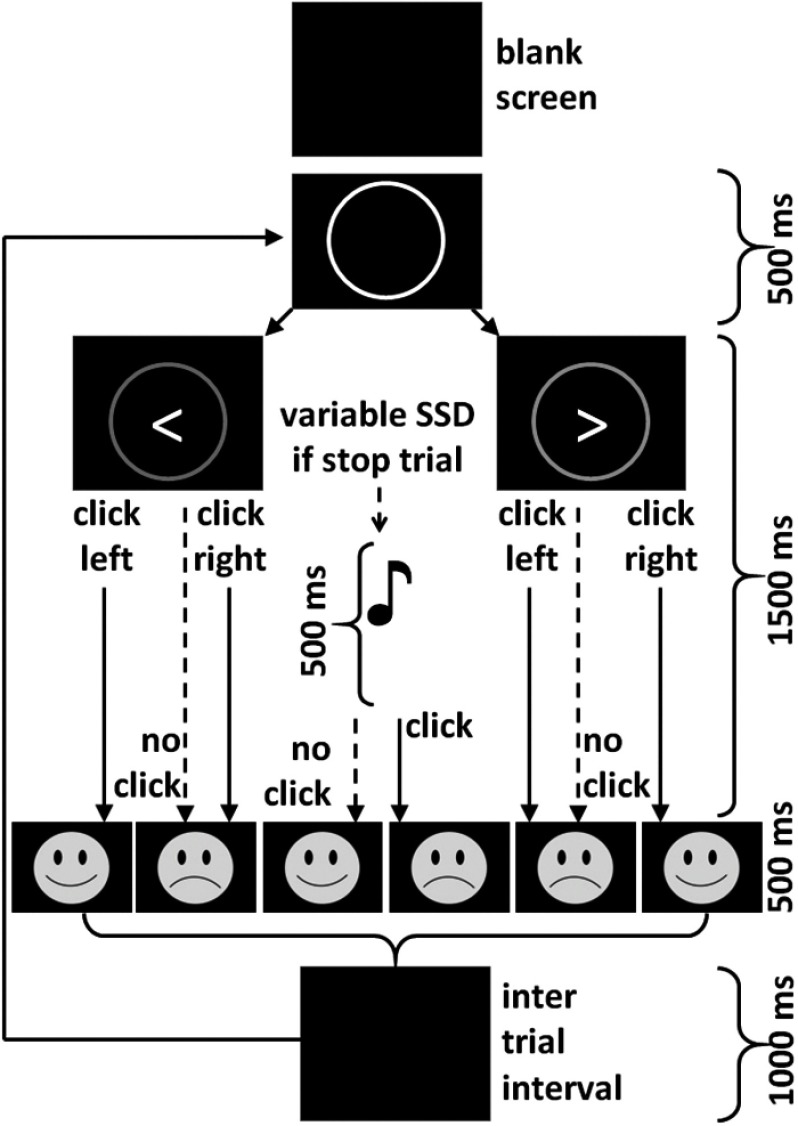
Events in the stop signal task. Each trial starts with a blank screen that turns into a white fixation circle. The fixation circle then turns green when the go signal (either left or right arrow) is presented. This is occasionally followed by a stop signal (auditory tone). Depending on the participant’s response, they were then presented with feedback of either a smiley or a frowney face as indicated. Trials were separated by a null time that ranged between 0.5 and 4 s (mean, 1 s; sampled from an exponential distribution truncated at 4 s) as in Aron and Poldrack (2006). *Source*: Modified figure and legend from Shadli et al. (2015), with permission.

The SST (see Figure 2) was based on Aron and Poldrak (2006) with some minor adjustments to the control of SSDs (McNaughton et al., 2013) and adjustments to the task for children made by Stevenson (2011).

Trials were of two types: Go; and Stop. All started with presentation of a fixation circle in the centre of the screen against a black background. After 500 ms, a Go stimulus appeared within the circle that was a left arrow (<) or a right arrow (>), to which participants were instructed to make a left or right click, respectively, as quickly as possible. If a response was made, or after 1500 ms, the circle and arrow disappeared. Stop trials were like Go trials except for a 1000 Hz tone, presented for 500 ms, requiring the participants to withhold responding. The SSDs varied systematically between trials. Participants were told that stopping and going were equally important.

The SSD on any particular stop trial was controlled by one of the four “staircases”, varied from trial to trial. Correct stopping to a particular staircase moved its SSD value up (harder), incorrect going moved its SSD value down (easier) in 50 ms steps. Except that, when the staircase delivered the mean SSD then the shift was by 100 ms rather than the usual 50 ms; this increased the spread of the SSD distribution and so the discrimination between the three different types of trial analysed.

Stevenson’s (2011) modifications for children were: (1) The SSD delivery at the start of each block was set to the mean SSD of the previous block rather than being an integer multiple of 50 ms (this increased the probability that intermediate SSDs would result in 50% correct stopping and so maximise conflict); (2) There were 6 half-blocks of 64 trials, instead of 3 uninterrupted blocks of 128 trials (and so 2 more break periods to aid concentration and reduce restlessness and so movement artefacts in the EEG) – 128-trial blocks were used for analysis as previously; (3) Four short humorous GIF clips were provided at each rest time with different clips for each of the six rest periods; (4) The maximum duration of Go stimulus presentation was 1500 ms (Stevenson found significant failure of typically developing children to complete the Go response within the typical adult 1000 ms time limit); and (5) After each trial, performance feedback was presented briefly (250 ms). Correct responding was signalled by a smiling face incorrect by a frowning face (Figure 2). The SST lasted 25–35 min depending on the length of rest breaks. As noted in results, the children generally did not complete the full adult sequence of trials (which is set to the maximum over which adults produce reliable responding).

### 1.4 Behavioural data analysis

For each trial, we recorded trial and block number, trial type (Go or Stop), SSD value, reaction time, staircase index (1–4), staircase moves for each stair case, physical response (left/right/null) and inter-trial intervals. Based on Aron and Poldark’s (2006) study, three measures of behaviour were derived: (1) the median reaction time (MRT) of Go trials (Go RT) across all trials in ms; (2) the mean SSD over the last 12 moves of the 4 staircases in ms; and (3) the Stop Signal Reaction Time (SSRT) in ms was calculated by subtracting the mean SSD from the median Go RT of each participant. The percentage of trials with successful inhibition (***P***_***inhibit***_) of the 4 staircases in the last 12 moves was calculated to verify that ***P***_***inhibit***_ had converged at 50%. The ***P***_***inhibit***_ was then calculated for each group of SSDs.

### 1.5 EEG data analysis

EEG data were processed using a purpose-built program in Visual Basic 6, as used in our previous adult studies that developed the biomarker to allow direct comparison. Data collected in the two studies were processed in New Zealand by the same experimenter. The raw data (128 Hz) were first low pass filtered (using a 3-point running mean, effective cut-off 43 Hz) to remove residual high frequency noise. Eye blink artefacts were removed by first fitting a ballistic template to Fp1 (Zhang et al., 2017) and then removing the fitted component from each channel after scaling with conventional least squares regression (Gratton, 1998) to leave residual EEG. Remaining artefacts associated with eye blinks, movement, etc. were manually replaced with missing values.

Raw EEG was Fast Fourier transformed and converted to the log power spectrum. Spectra focussed on the 0.5-s period after the tone in Stop trials and on the same 0.5 s period in the preceding Go trial (or, if the preceding trial was a stop trial, then the following Go trial). Each analysed epoch consisted of 32 samples before presentation of the 500 ms tone, 64 samples during presentation, and 32 samples after the end of the tone. We applied a Hanning window (a cosine wave which extracts most power from the middle 0.5 s and little from the two outlying 0.25 ms), which improves transform quality and doubles frequency resolution (to 1 Hz) compared to an equivalent (0.5 s) square window. A log transform then normalised error variance before averages was calculated.

Correlation of average SSDs from the first and second staircases with those of the third and fourth staircases tested for stability of SSD values. The 50% probability of withholding or responding had stabilised by the last 12 changes of each staircase. Trials were groups by SSD values that varied the level of conflict. For each participant, 48 Stop trials (4 staircases × last 12 changes) were arranged in the order of ascending SSD and then divided into early, intermediate and late groups. Trials with the same SSD were always put in the same group with unequal numbers of trials in the different groups for some participants. The number of trials in each band differed from 5 to 24 before averaging.

The last 12 trials of each staircase (when ***P***_***inhibit***_ converged at 50%) were taken and the spectra for early, intermediate and late SSDs were averaged separately. Any missing data in the window resulted in the entire spectrum for that period of EEG being marked missing. When more than 30% of the trials contributing to an average of spectral power were missing, the average was replaced with missing data markers. Participants were excluded from the analysis when more than 10% of their overall data were missing.

### 1.6 Statistical analysis – analysis of variance (ANOVA)

ANOVAs were performed with IBM SPSS package 21. For EEG analysis, group factors were country (NZ, Iran) and diagnosis (control, ADHD-C, ADHD-I), and repeated measures were SSD (early, intermediate and late), frequency (4–12 Hz), and trial type (Stop and Go).

The GCSR component was calculated as follows. The intermediate SSD is where maximum conflict occurs between stopping and going. Late and early SSDs represent lower levels of conflict but higher or lower levels of other aspects of the task such as stopping. The linear and quadratic contrasts of the three levels of SSD allow separation of these two types of effect. For GCSR, the conflict-specific component is extracted as the orthogonal quadratic contrast of SSD. This component is identical to the difference between the intermediate SSD and the average of the short and long SSDs and so the term “quadratic” is descriptive and does not imply the presence of an underlying quadratic function. We also ensure that the difference is specific to the occurrence of the stop signal by testing the difference between the two types of trial (treated by SPSS as a linear trend). So effects common to go and stop trials are eliminated. The EEG statistics reported below are limited to analysis of GCSR (calculated as the type[lin] × SSD[quad] component by SPSS). The resultant F ratios are the same as would have been obtained if we had first calculated GCSR and then undertaken ANOVA and so have been reported in this form for simplicity. Note also that effects of frequency were determined via orthogonal polynomial coefficients (with 1 frequency df) eliminating the sphericity problem normally encountered with repeated measures.

## 2. Results

### 2.1 Behavioural measures

More than half of the participants did the task only up to the end of block 2 (i.e. 512 trials) and did not wish to proceed to block 3. In addition, all participants showed stabilised SSDs in block 2, even those who completed block 3. SSDs were not stable in block 1 as this is a learning period. Behavioural and EEG analysis, therefore, focussed on block 2 to maximise the number of participants included. ANOVAs were carried out on each of MRT, SSRT, Go correct%, and Probability of Inhibition (***P***_***inhibit***_) in short, intermediate and long SSD testing for differences between the three diagnosed groups (control, ADHD inattentive, and ADHD combined) and the two genders. The data are presented in Table 1.

**Table 1.** Behaviour divided by gender and diagnostic group

A: Initial study (New Zealand)												
	Female						Male					
	Control		Inattentive		Combined		Control		Inattentive		Combined	
MRT (SD) ms	587	(42)	633	(55)	610	(46)	588	(56)	575	(51)	569	(61)
SSRT (SD) ms	287	(79)	291	(43)	330	(6)	210	(32)	353	(76)	372	(50)
Go correct (SD)%	94	(2)	91	(6)	95	(3)	95	(2)	86	(10)	83	(8)
***P***_***inhibit***_ short(SD)%	75	(6)	95	(11)	79	(11)	78	(5)	64	(6)	72	(5)
***P***_***inhibit***_ intermediate(SD)%	56	(5)	56	(8)	55	(8)	60	(4)	53	(4)	52	(4)
***P***_***inhibit***_ long(SD)%	36	(5)	37	(9)	40	(9)	38	(4)	37	(4)	41	(4)
N	6		2		2		9		7		8	
B: Main study (Iran)												
MRT (SD) ms	613	(61)	635	(93)	627	(32)	597	(46)	611	(67)	560	(71)
SSRT(SD) ms	351	(97)	407	(99)	326	(67)	269	(45)	321	(66)	300	(89)
Go correct (SD)%	88	(8)	70	(16)	73	(31)	92	(4)	87	(8)	81	(20)
***P***_***inhibit***_ short(SD)%	70	(18)	67	(13)	74	(16)	75	(7)	71	(13)	64	(11)
***P***_***inhibit***_ intermediate(SD)%	53	(10)	49	(6)	50	(9)	52	(6)	55	(13)	51	(9)
***P***_***inhibit***_ long(SD)%	36	(10)	36	(11)	42	(15)	36	(11)	37	(10)	36	(17)
N	17		7		8		10		11		13	

MRT = Median Reaction Time; SSRT = Stop Signal Reaction Time; Go Correct% = the Percentage of Correct Responses on Go Trials; ***P***_***inhibit***_ Short = Probability of Correct Response on Stop Trials with Short SSDs; ***P***_***inhibit***_ Intermediate = Probability of Correct Response on Stop Trials with Intermediate SSDs; ***P***_***inhibit***_ Long = Probability of Correct Response on Stop Trials with Long SSDs; N = Number of Participants in Each Group.

In the initial study (Table 1A), there were no significant variations between the groups in MRT, Go correct% and ***P***_***inhibit***_ measures. However, both ADHD subtypes appeared to show longer SSRTs compared to the control group particularly in males (diagnosis: F(2, 28) = 8.208, *p* < .01; diagnosis × gender: F(2, 28) = 4.227, *p* < .05). Nevertheless, the two genders did not differ when pooled across diagnosis (gender: F(1, 28) = 0.140, *p* = .712).

In the main study (Table 1B), we observed somewhat different results. There were no significant differences between diagnostic groups in MRT, SSRT and ***P***_***inhibit***_ measures. However, ADHDs showed reliably lower Go correct% than controls (diagnosis: F(2, 60) = 4.364, *p* < .05).

Given the weak, site-specific, gender effects and the smaller *N* with subdivision by gender, the EEG data were analysed pooled across gender.

### 2.2 EEG analysis

**Figure 3.**
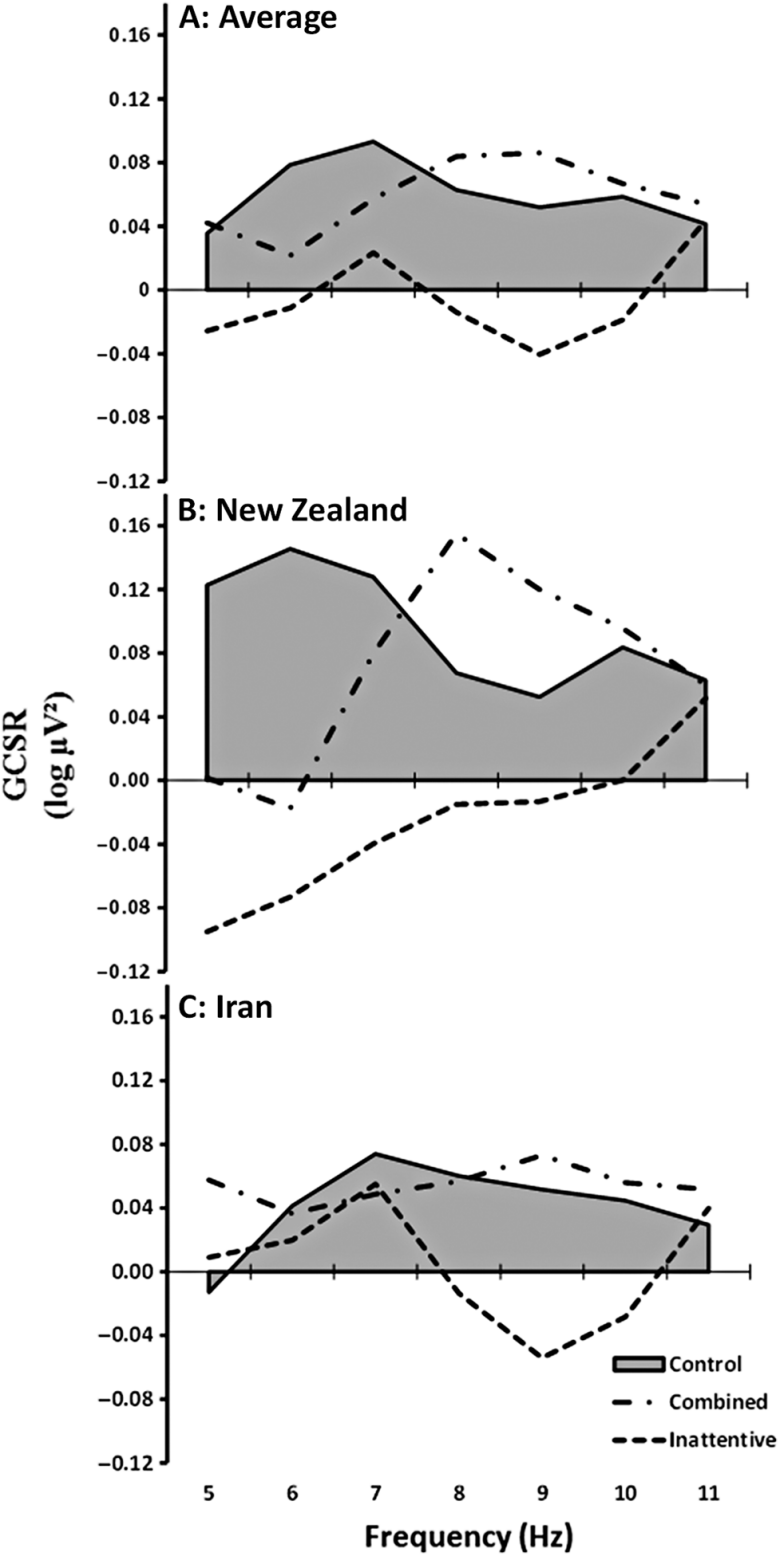
Variation in goal-conflict-specific rhythmicity (GCSR) at F8 for each frequency averaged across all participants for the three diagnostic groups (control, combined and inattentive subtype) for Block 2 of the SST. The data were smoothed with a 3-point running average for display but not analysis. (A) an average of the results from the two centres. (B) results of initial study (New Zealand). (C) results of main study (Iran).

Overall (Figure 3A), control participants showed positive GCSR particularly in the region of 6–7 Hz. This confirms the preliminary observation of GCSR in children by Stevenson (2011). Similarly, the ADHD-C showed positive GCSR across all frequencies, but peaking at 8–9 Hz. In contrast, the ADHD-I mostly showed negative GCSR. The ADHD-I pattern was clearly distinct from ADHD-C particularly across the frequency range of 8–10 Hz.

While these results were qualitatively similar across the two studies (Figure 3B, C), the combined effects were not significant as there were apparent quantitative differences in the detailed shape of the curves. The curves of the different groups peaked in somewhat different places with the result that the difference between countries in the differences between groups appeared to have multiple peaks (country × group × frequency[order 6], F(2,92) = 3.146, *p* < .05, ηp^2^ = 7%; country × group × frequency[order 5], F(2,92) = 2.083, *p* = .13, ηp^2^ = 4%). These were explored with post hoc ANOVAs.

For post hoc analysis restricted to the initial NZ study, the three groups differed on the order 6 component distinguishing the two studies (group × frequency[order 6], F(2,29) = 3.849, *p* < .05, ηp^2^ = 21%). As can be seen in Figure 3B, ADHD-C had high GCSR activity across 8–10 Hz compared to controls, who had high activity at 5–7 Hz (post hoc [ADHD-C vs. Control] × frequency[order 6], F(1,21) = 6.085, *p* < .05, ηp^2^ = 22%; [ADHD-C vs. Control] × frequency[order 4], F(1,21) = 7.222, *p* < .05, ηp^2^ = 26%). In contrast, ADHD-I had negative GCSR across the 5–9 Hz frequency range but this group was not reliably different from either ADHD-C or Control on post hoc ANOVA (post hoc [ADHD-I vs. Control] × frequency[order 6], F(1,21) = 1.215, *p* = .282; post hoc [ADHD-I vs. ADHD-C] × frequency[order 6], F(1,21) = 3.778, *p* = .071).

For post hoc analysis, restricted to the main Iran study, the three groups differed on the order 5 component that approached a significant difference between the two studies (group × frequency[order 5], F(2,63) =4.432, *p* < .05, ηp^2^ = 12%). As seen in Figure 3C, ADHD-C had a somewhat similar GCSR pattern in the 5–10 Hz frequency range to the control group (post hoc [ADHD-C vs. Control] × frequency[order 5], F(1,46) = 1.331,*p* = .255). In contrast, ADHD-I appeared to have a distinct pattern from the other groups with a noticeable decrease in power at 8–10 Hz but a tendency to positive values at lower frequencies (5–8 Hz). Post hoc ANOVA found a clear difference between ADHD-C and ADHD-I (post hoc [ADHD-C vs. ADHD-I] × frequency[order 5], F(1,37) = 8.167, *p* = .007, ηp^2^ = 18%) and a less reliable difference between ADHD-I and controls (post hoc [ADHD-I vs. controls] × frequency[order 5], F(1,43) = 4.163, *p* < .05, ηp^2^ = 9%).

Overall, then, there is only moderate evidence that GCSR of ADHD-C is different from controls. Critically, in both studies, ADHD-C do not have lower values than controls and, unlike ADHD-I, generally do not have negative values in the 5–10 Hz range. The differences between the two sets of results, and complex shapes observed, suggest that the specific shapes of the ADHD curves in Figure 3A may not be replicable.

## 3. Discussion

### 3.1 Overview

We found solid evidence in children of positive GCSR, of the type previously observed in adults, for the control and ADHD-C groups. There were signs of lower GCSR activity for the ADHD-I subtype. Importantly, GCSR at F8 for the control and ADHD-C subtype occurred in both studies and so detection in children of GCSR as a qualitative result is robust in the face of differences in geography, race, culture, size of urban area, recruitment method and diagnostician. Qualitatively there were signs of reduction in GCSR for the ADHD-I group in both experiments. Hence, our BIS biomarker may differentiate, at least on a group basis, between the EEG power of the subtypes (ADHD-I, ADHD-C) but provides no evidence of a reduction of GCSR in ADHD-C relative to control.

We found the predicted reduction in our BIS biomarker for ADHD-I, supporting Quay’s (1997) view that ADHD symptoms might be due to underactivity in the BIS. However, contrary to prediction, there was no evidence of reduced BIS activity for ADHD-C. The current ADHD-C results are also potentially inconsistent with Barkley’s (1997b) hierarchical model of impairments in ADHD. In Barkley’s model, inhibition deficits are primary and lead to secondary impairments in the other four neuropsychological areas. This is not consistent with our ADHD-C data if “behavioural inhibition” is restricted to the sense of BIS (goal conflict) output as opposed to motor stopping. Normal, or perhaps enhanced, BIS activity for the ADHD-C subtype is more consistent with Nigg’s (2006) suggestion that hyperactive-impulsive behaviours (which are more common in ADHD-C than ADHD-I) are an expression of a high approach tendency (i.e. high BAS sensitivity in RST terms) rather than behavioural inhibition impairments. Solanto et al. (2001) also suggested that impulsivity in ADHD reflects more a choice to avoid delay than an inability to inhibit responses; and that delay aversion is associated with a broader range of ADHD characteristics than is disinhibition.

SSRT has been widely seen as the essential measure characterising behavioural inhibition performance in ADHD. However, a recent meta-analysis by Alderson, Rapport and Kofler (2007) argued that SSRT reflects a more generalised deficit in cognitive processing rather than behavioural inhibition. In the current research, ADHD subtypes showed longer SSRTs than the control groups across the two studies with the sole exception that the ADHD-C female group in the main study showed slightly shorter SSRTs than the control group. This finding of longer SSRTs for ADHD groups is consistent with the previous studies (Alderson, Rapport, Sarver, & Kofler, 2008; Lipszyc & Schachar, 2010) which showed significantly slower and more variable SSRTs for ADHD groups. Longer SSRTs in children with ADHD have been interpreted as proof for BIS deficits (Quay, 1997). However, it should be noted that, in two variants of the current task, SSRT was not affected by anxiolytic drugs despite their reduction of GCSR (McNaughton et al., 2013; Shadli et al., 2015). Similarly, GCSR did not predict SSRT in either of those studies nor in Neo et al. (2011) and there is no correlation of SSRT with trait anxiety, or neuroticism (Neo et al., 2011).

Neo et al. (2011) suggested that stopping in the SST is a speeded response task involving acts (Floden & Stuss, 2006) or actions (Aron & Poldrack, 2006), with the detection of goal conflict involving distinct limbic circuits (Figure 1) that are too slow to affect stopping behaviour in the SST. On this view, ADHD could involve problems with relatively fast action stopping controlled by the right inferior frontal gyrus (Aron & Poldrack, 2006) in both ADHD-I and ADHD-C (Rubia, Cubillo et al., 2010; Rubia, Halari et al., 2010) but problems with conflict processing (and hence arousal, attention, and behavioural inhibition in the sense defined by Gray for the BIS) only in ADHD-I. The fast action stopping problems are likely linked to the ADHD deficit seen in the N2 potential (Albrecht et al., 2005; Dimoska et al., 2003; Pliszka et al., 2000), which occurs in the region of 200 ms into the stop signal delivery and is fast enough to be functionally involved in stopping.

ADHD subtypes were not distinguishable from the typically developing groups in terms of the Go response speed in either the initial study or the main study. Previous studies have found contradictory MRT results for children with ADHD. According to Alderson et al. (2007), ADHD groups had significantly slower MRT; but de Zeeuw et al. (2008) showed ADHD groups had reliably faster MRT. We found more inaccurate responses in Go trials for ADHDs than controls in the main study, which fits with the results of de Zeeuw et al. (2008).

In sum, some ADHD-I symptoms, which involve attention and arousal problems, may be explained by low BIS sensitivity. However, ADHD-C do not appear to differ from controls in terms of BIS sensitivity, contrary to our predictions and despite apparently similar abnormalities to ADHD-I in BIS-related structures (Corr & McNaughton, 2016). ADHD-C symptoms (and some ADHD-I symptoms), which are characterised by impulsive behaviours, may be better explained by abnormality in action stopping, approach systems, or other executive functions controlled by prefrontal cortex and linked to dopaminergic abnormalities (Sonuga-Barke, 2005).

### 3.2 Limitations

Quantitative aspects of the current results are preliminary. Recruitment problems resulted in a relatively small sample of participants in the initial study (New Zealand). Sample size is particularly important for the interpretation of the variation in GCSR with frequency between studies as this function appears variable (compare McNaughton et al., 2013; Neo et al., 2011). There were not equal numbers of both genders in each diagnostic group because of the limited number of participants in both the initial and the main studies. As a result, we could not control gender effects across subtypes. We also did not screen for and exclude on comorbid factors, such as behavioural problems or learning disabilities. Much larger samples of each subtype group, including equal numbers of each gender and the exclusion of comorbidities, would be desirable.

The sampled populations of the two studies differed in terms of diagnostic environment, diagnosing clinicians, culture, and race. Moreover, somewhat different EEG hardware and software were used in the initial study (ASA Neurotechnology) and in the main study (WinEEG) for EEG recording. Two different kinds of EEG caps were used in each centre (the initial study: silver/silver chloride electrode cap; main study: tin electrode cap). All these uncontrolled variables could impact the final results. However, given the overall qualitative consistency of the results obtained, these uncontrolled differences make our overall conclusions stronger by demonstrating generality across the varying conditions.

Finally, it should be noted that our BIS biomarker should, theoretically, represent overall sensitivity of the whole BIS – a hierarchical system spanning from the periaqueductal grey to the prefrontal cortex. While changes in it confirm overall BIS involvement in a process, it does not exclude the possibility that sensitivity of discrete parts of the BIS (e.g. the amygdala) could underlie some facets of externalising in general and ADHD in particular.

### 3.3 Conclusions

Is BIS dysfunction the *core* of all ADHD symptoms as previously hypothesised (Barkley, 1997a; Quay, 1997)? Our findings suggest that the answer to this question is “no”. Averaged GCSR activity was consistently high for the ADHD-C and the control groups in both studies. These results raise the question of whether low BIS sensitivity contributes to other externalising conditions, as previously proposed (Corr & McNaughton, 2016; Fowles, 1980).

Is low BIS sensitivity maladaptive? We found some aspects of ADHD symptoms that could relate to BIS dysfunction. In particular, averaged GCSR activity, the BIS biomarker, tended to be consistently lower for the ADHD-I groups across the two studies. Thus, ADHD-I symptoms such as low levels of attention and arousal could be due to BIS under activity since these are BIS outputs. We conclude that ADHD-I differ from ADHD-C and healthy groups in terms of their BIS sensitivity.

However, we cannot explain action-stopping problems in ADHD-C by low BIS sensitivity as there was no evidence of this for ADHD-C in either of the studies and, critically, action stopping is not sensitive to the anxiolytic drugs that define the BIS. Rather than BIS under activity, BAS over activity may better explain ADHD-C hyperactivity and impulsivity symptoms. Future work is needed to determine if two different motivational systems are involved in ADHD-I and ADHD-C symptoms: (1) a low BIS sensitivity in ADHD-I, which causes goal conflict resolution problems; and (2) a high BAS sensitivity in ADHD-C, which causes action stopping problems.

BIS dysfunction may then contribute to some aspects of ADHD and to some differences between the subtypes but other prefrontal systems and contributions from, e.g., dopamine, are likely to provide significant additional contributions. The same is likely to be true for the whole externalising spectrum (Beauchaine et al., 2018).

## References

[ref1] Albrecht, B. , Banaschewski, T. , Brandeis, D. , Heinrich, H. , & Rothenberger, A. (2005). Response inhibition deficits in externalizing child psychiatric disorders: An ERP-study with the Stop-task. Behavioral and Brain Functions, 1, A22 10.1186/1744-9081-1-22. PMC1343568 16336676

[ref2] Albrecht, B. , Brandeis, D. , Uebel, H. , Valko, L. , Heinrich, H. , Drechsler, R. , … Banaschewski, T. (2013). Familiality of neural preparation and response control in childhood attention deficit-hyperactivity disorder. Psychological Medicine, 43, 1997–2011. 10.1017/S003329171200270X. 23200032

[ref3] Alderson, R. M. , Rapport, M. D. , & Kofler, M. J. (2007). Attention-deficit/hyperactivity disorder and behavioral inhibition: A meta-analytic review of the stop-signal paradigm. Journal of Abnormal Child Psychology, 35, 745–758. 10.1007/s10802-007-9131-6. 17668315

[ref4] Alderson, R. M. , Rapport, M. D. , Sarver, D. E. , & Kofler, M. J. (2008). ADHD and behavioral inhibition: A re-examination of the stop-signal task. Journal of Abnormal Child Psychology, 36, 989–998. 10.1007/s10802-008-9230-z. 18461439

[ref5] Andrews, G. , Stewart, G. , Morris-Yates, A. , Holt, P. , & Henderson, S. (1990). Evidence for a general neurotic syndrome. British Journal of Psychiatry, 157, 6–12. 10.1192/bjp.157.1.6. 2397364

[ref6] Aron, A. R. , & Poldrack, R. A. (2006). Cortical and subcortical contributions to stop signal response inhibition: Role of subthalamic nucleus. The Journal of Neuroscience, 26, 2424–2433. 10.1523/JNEUROSCI.4682-05.2006. 16510720 PMC6793670

[ref7] Baeyens, D. , Roeyers, H. , & Walle, J. V. (2006). Subtypes of attention-deficit/hyperactivity disorder (ADHD): Distinct or related disorders across measurement levels? Child Psychiatry Human Development, 36, 403–417. 10.1007/s10578-006-0011-z. 16755403

[ref8] Barkley, R. A. (1997a). Attention deficit/hyperactivity disorder, self-regulation, and time: Toward a more comprehensive theory. Journal of Developmental and Behavioral Paediatrics, 18, 271–279. 10.1097/00004703-199708000-00009. 9276836

[ref9] Barkley, R. A. (1997b). Behavioral inhibition, sustained attention, and executive functions: Constructing a unifying theory of ADHD. Psychological Bulletin, 121, 65–94. 10.1037/0033-2909.121.1.65. 9000892

[ref10] Barkley, R. A. , Grodzinsky, G. , & DuPaul, G. J. (1992). Frontal lobe functions in attention deficit disorder with and without hyperactivity: A review and research report. Journal of Abnormal Child Psychology, 20, 163–188. 10.1007/bf00916547. 1593025

[ref11] Beauchaine, T. P. , & McNulty, T. (2013). Comorbidities and continuities as ontogenic processes: Toward a developmental spectrum model of externalizing psychopathology. Development and Psychopathology, 25, 1505–1528. 10.1017/S0954579413000746. 24342853 PMC4008972

[ref12] Beauchaine, T. P. , McNulty, T. , & Hinshaw, S. P. (2016). An ontogenic processes model of externalizing psychopathology In T. P. Beauchaine & S. P. Hinshaw (Eds.), The Oxford handbook of externalizing spectrum disorders (pp. 485–501). New York: Oxford University Press.

[ref13] Beauchaine, T. P. , Zisner, A. R. , & Hayden, E. P. (2018). Neurobiological mechanisms of psychopathology and treatment action In T. H. Ollendick , S. W. White , & B. A. White (Eds.), The Oxford handbook of clinical child and adolescent psychology. Oxford Handbooks Online: Oxford University Press 10.1093/oxfordhb/9780190634841.013.54.

[ref14] Cloninger, C. R. , Svrakic, D. M. , & Przybecky, T. R. (1993). A psychobiological model of temperament and character. Archives of General Psychiatry, 50, 975–990. 10.1001/archpsyc.1993.01820240059008. 8250684

[ref15] Coghill, D. R. , Hayward, D. , Rhodes, S. M. , Grimmer, C. , & Matthews, K. (2014). A longitudinal examination of neuropsychological and clinical functioning in boys with attention deficit hyperactivity disorder (ADHD): Improvements in executive functioning do not explain clinical improvement. Psychological Medicine, 44, 1087–1099. 10.1017/S0033291713001761. 23866120

[ref16] Conners, C. K. , Sitarenios, G. , Parker, J. D. A. , & Epstein, J. N. (1998). Revision and restandardization of the Conners Teacher Rating Scale (CTRS-R): Factor structure, reliability, and criterion validity. Journal of Abnormal Child Psychology, 26, 279–291. 10.1023/A:1022606501530. 9700520

[ref17] Corr, P. J. (Ed.) (2008). The reinforcement sensitivity theory of personality. Cambridge: Cambridge University Press.

[ref18] Corr, P. J. , & McNaughton, N. (2016). Neural mechanisms of low trait anxiety and risk for externalizing behaviour In T. P. Beauchaine & S. P. Hinshaw (Eds.), The Oxford handbook of externalizing spectrum disorders (pp. 220–238). Oxford: Oxford University Press 10.1093/oxfordhb/9780199324675.013.1.

[ref19] Cortese, S. , Kelly, C. B. , Chabernaud, C. , Proal, E. , Di Martino, A. , Milham, M. P. , … Castellanos, F. X. (2012). Toward systems neuroscience of ADHD: A meta-analysis of 55 fMRI studies. American Journal of Psychiatry, 169, 1038–1055. 10.1176/appi.ajp.2012.11101521. 22983386 PMC3879048

[ref20] de Zeeuw, P. , Aarnoudse-Moens, C. , Bijlhout, J. , Konig, C. , Uiterweer, A. P. , Papanikolau, A. , … Oosterlaan, J. (2008). Inhibitory performance, response speed, intraindividual variability, and response accuracy in ADHD. Journal of the American Academy of Child and Adolescent Psychiatry, 47, 808–816. 10.1097/CHI.0b013e318172eee9. 18520957

[ref21] DeYoung, C. G. , & Krueger, R. F. (2018). A cybernetic theory of psychopathology. Psychological Inquiry, 29, 117–138. 10.1080/1047840X.2018.1513680.

[ref22] Dimoska, A. , Johnstone, S. J. , Barry, R. J. , & Clarke, A. R. (2003). Inhibitory motor control in children with attention-deficit/hyperactivity disorder: Event-related potentials in the stop-signal paradigm. Biological Psychiatry, 54, 1345–1354. 10.1016/S0006-3223(03)00703-0. 14675798

[ref23] Elliot, A. J. , & Thrash, T. M. (2002). Approach-avoidance motivation in personality: Approach and avoidance temperament and goals. Journal of Personality and Social Psychology, 82, 804–818. 10.1037//0022-3514.82.5.804. 12003479

[ref24] Elliot, A. J. , & Thrash, T. M. (2010). Approach and avoidance temperament as basic dimensions of personality. Journal of Personality, 78, 865–906. 10.1111/j.1467-6494.2010.00636.x. 20573129

[ref25] Eysenck, H. J. , & Eysenck, S. B. G. (1964). Eysenck personality inventory. London: University of London Press.

[ref26] Floden, D. , & Stuss, D. T. (2006). Inhibitory control is slowed in patients with right superior medial frontal damage. Journal of Cognitive Neuroscience, 18, 1843–1849. 10.1162/jocn.2006.18.11.1843. 17069475

[ref27] Fowles, D. C. (1980). The three arousal model: Implications of Gray’s two-factor learning theory for heart rate, electrodermal activity and psychopathy. Psychophysiology, 17, 87–104. 10.1111/j.1469-8986.1980.tb00117.x. 6103567

[ref28] Gomez, R. , Woodworth, R. , Waugh, M. , & Corr, P. J. (2012). Attention-deficit/hyperactivity disorder symptoms in an adult sample: Associations with Cloninger’s temperament and character dimensions. Personality and Individual Differences, 52, 290–294. 10.1016/j.paid.2011.10.015.

[ref29] Gratton, G. (1998). Dealing with artifacts: EOG contamination of the event-related brain potential. Behavior Research Methods, 30, 44–53. 10.3758/BF03209415.

[ref30] Gray, J. A. (1970). The psychophysiological basis of introversion-extraversion. Behaviour Research and Therapy, 8, 249–266. 10.1016/0005-7967(70)90069-0. 5470377

[ref31] Gray, J. A. (1972). The psychophysiological nature of introversion-extraversion: A modification of Eysenck’s theory In V. D. Nebylitsyn & J. A. Gray (Eds.), The biological bases of individual behaviour (pp. 182–205). London: Academic Press.

[ref32] Gray, J. A. (1977). Drug effects on fear and frustration: Possible limbic site of action of minor tranquilizers In L. L. Iversen , S. D. Iversen , & S. H. Snyder (Eds.), Handbook of psychopharmacology. Vol 8. Drugs, neurotransmitters and behaviour (pp. 433–529). New York: Plenum Press.

[ref33] Gray, J. A. (1982). The neuropsychology of anxiety: An enquiry in to the functions of the septo-hippocampal system. Oxford: Oxford University Press.

[ref34] Gray, J. A. , & McNaughton, N. (2000). The neuropsychology of anxiety: An enquiry into the functions of the septo-hippocampal system (2nd ed.). Oxford: Oxford University Press.

[ref35] Kaufman, J. , Birmaher, B. , Brent, D. , Rao, U. M. A. , Flynn, C. , Moreci, P. , … Ryan, N. (1997). Schedule for affective disorders and schizophrenia for school-age children-present and lifetime version (K-SADS-PL): Initial reliability and validity data. Journal of the American Academy of Child & Adolescent Psychiatry, 36, 980–988. 10.1097/00004583-199707000-00021. 9204677

[ref36] Krueger, R. F. , Hicks, B. M. , Patrick, C. J. , Carlson, S. R. , Iacono, W. G. , & McGue, M. (2002). Etiologic connections among substance dependence, antisocial behavior and personality: Modeling the externalizing spectrum. Journal of Abnormal Psychology, 111, 411–424. 10.1037/0021-843X.111.3.411. 12150417

[ref37] Lemiere, J. , Wouters, H. , Sterken, C. , Lagae, L. , Sonuga-Barke, E. , & Danckaerts, M. (2010). Are children with ADHD predominantly inattentive and combined subtypes different in terms of aspects of everyday attention? Europena Child and Adolescent Psychiatry, 19, 679–685. 10.1007/s00787-010-0105-9. 20361222

[ref38] Lipszyc, J. , & Schachar, R. (2010). Inhibitory control and psychopathology: A meta-analysis of studies using the stop signal task. Journal of the International Neuropsychological Society, 16, 1064–1076. 10.1017/S1355617710000895. 20719043

[ref39] McNaughton, N. (2018). What do you mean “anxiety”? Developing the first anxiety syndrome biomarker. Journal of the Royal Society of New Zealand, 48, 177–190. 10.1080/03036758.2017.1358184.

[ref40] McNaughton, N. , & Corr, P. J. (2008). RST and personality In P. J. Corr (Ed.), The reinforcement theory of personality (pp. 155–187). Cambridge: Cambridge University Press.

[ref41] McNaughton, N. , & Sedgwick, E. M. (1978). Reticular stimulation and hippocampal theta rhythm in rats: Effects of drugs. Neuroscience, 2, 629–632. 10.1016/0306-4522(78)90004-0. 724111

[ref42] McNaughton, N. , Kocsis, B. , & Hajos, M. (2007). Elicited hippocampal theta rhythm: A screen for anxiolytic and procognitive drugs through changes in hippocampal function? Behavioural Pharmacology, 18, 329–346. Retrieved from http://www.ncbi.nlm.nih.gov/pubmed/17762505 17762505 10.1097/FBP.0b013e3282ee82e3

[ref43] McNaughton, N. , Swart, C. , Neo, P. , Bates, V. , & Glue, P. (2013). Anti-anxiety drugs reduce conflict-specific “theta”–a possible human anxiety-specific biomarker. Journal of Affective Disorders, 148, 104–111. 10.1016/j.jad.2012.11.057. 23261140

[ref44] Miller, D. J. , Derefinko, K. J. , Lynam, D. R. , Milich, R. , & Fillmore, M. T. (2010). Impulsivity and attention deficit-hyperactivity disorder: Subtype classification using the UPPS impulsive behavior scale. Journal of Psychopathology and Behavioral Assessment, 32, 323–332. 10.1007/s10862-009-9155-z. 21765593 PMC3137261

[ref45] Mullins-Sweatt, S. N. , & Lengel, G. J. (2012). Clinical utility of the five-factor model of personality disorder. Journal of Personality, 80, 1615–1639. 10.1111/j.1467-6494.2012.00774.x. 22321379

[ref46] Neo, P. S. H. , Thurlow, J. , & McNaughton, N. (2011). Stopping, goal-conflict, trait anxiety and frontal rhythmic power in the stop-signal task. Cognitive Affective & Behavioral Neuroscience, 11, 485–493. 10.3758/s13415-011-0046-x. 21647572

[ref47] Nichols, S. L. , & Waschbusch, D. A. (2004). A review of the validity of laboratory cognitive tasks used to assess symptoms of ADHD. Child Psychiatry Human Development, 34, 297–315. 10.1023/B:CHUD.0000020681.06865.97. 15039603

[ref48] Nigg, J. T. (2006). Temperament and developmental psychopathology. Journal of Child Psychology and Psychiatry, 47, 395–422. 10.1111/j.1469-7610.2006.01612.x. 16492265

[ref49] Penke, L. , Denissen, J. J. A. , & Miller, G. F. (2007). The evolutionary genetics of personality. European Journal of Personality, 21, 549–587. 10.1002/per.629.

[ref50] Pliszka, S. R. , Liotti, M. , & Woldorff, M. G. (2000). Inhibitory control in children with attention-deficit/hyperactivity disorder: Event-related potentials identify the processing component and timing of an impaired right-frontal response-inhibition mechanism. Biological Psychiatry, 48, 238–246. 10.1016/S0006-3223(00)00890-8. 10924667

[ref51] Polanczyk, G. , de Lima, M. S. , Horta, B. L. , Biederman, J. , & Rohde, L. A. (2007). The worldwide prevalence of ADHD: A systematic review and metaregression analysis. American Journal of Psychiatry, 164, 942–948. 10.1176/ajp.2007.164.6.942. 17541055

[ref52] Quay, H. C. (1997). Inhibition and attention deficit hyperactivity disorder. Journal of Abnormal Child Psychology, 25, 7–13. 10.1177/108705479700200315. 9093895

[ref53] Rubia, K. , Cubillo, A. , Smith, A. B. , Woolley, J. , Heyman, I. , & Brammer, M. J. (2010). Disorder-specific dysfunction in right inferior prefrontal cortex during two inhibition tasks in boys with attention-deficit hyperactivity disorder compared to boys with obsessive–compulsive disorder. Human Brain Mapping, 31, 287–299. 10.1002/hbm.20864. 19777552 PMC6870854

[ref54] Rubia, K. , Halari, R. , Cubillo, A. , Mohammad, A. M. , Scott, S. , & Brammer, M. (2010). Disorder-specific inferior prefrontal hypofunction in boys with pure attention-deficit/hyperactivity disorder compared to boys with pure conduct disorder during cognitive flexibility. Human Brain Mapping, 31, 1823–1833. 10.1002/hbm.20975. 20205245 PMC6870768

[ref55] Sellbom, M. (2016). Elucidating the validity of the externalizing spectrum of psychopathology in correctional, forensic, and community samples. Journal of Abnormal Psychology, 125, 1027–1038. 10.1037/abn0000171. 27819465

[ref56] Shadli, S. M. , McIntosh, J. , Glue, P. , & McNaughton, N. (2015). An improved human anxiety process biomarker: Characterisation of frequency band, personality, and pharmacology. Translational Psychiatry, 5, e699 10.1038/tp.2015.188. 26670284 PMC5068587

[ref57] Sharma, A. , & Couture, J. (2014). A review of the pathophysiology, etiology, and treatment of Attention-Deficit Hyperactivity Disorder (ADHD). Annals of Pharmacotherapy, 48, 209–225. 10.1177/1060028013510699. 24259638

[ref58] Solanto, M. V. , Abikoff, H. , Sonuga-Barke, E. , Schachar, R. , Logan, G. D. , Wigal, T. , … Turkel, E. (2001). The ecological validity of delay aversion and response inhibition as measures of impulsivity in AD/HD: A supplement to the NIMH multimodal treatment study of AD/HD. Journal of Abnormal Child Psychology, 29, 215–228. 10.1023/a:1010329714819. 11411784

[ref59] Sonuga-Barke, E. J. S. (2005). Causal models of attention-deficit/hyperactivity disorder: From common simple deficits to multiple developmental pathways. Biological Psychiatry, 57, 1231–1238. 10.1016/j.biopsych.2004.09.008. 15949993

[ref60] Stevenson, M. (2011). Do phenylketonuria and attention deficit/hyperactivity disorder share a common dysfunction? A “behavioural inhibition system” hypothesis (MSc). Dunedin: University of Otago.

[ref61] Strelau, J. , & Zawadzki, B. (2011). Fearfulness and anxiety in research on temperament: Temperamental traits are related to anxiety disorders. Personality and Individual Differences, 50, 907–915. 10.1016/j.paid.2010.07.008.

[ref62] Trull, T. J. (2012). The five-factor model of personality disorder and DSM-5. Journal of Personality, 80, 1697–1720. 10.1111/j.1467-6494.2012.00771.x. 22321203

[ref63] Xia, S. , Foxe, J. J. , Sroubek, A. E. , Branch, C. , & Li, X. (2014). Topological organization of the “small-world” visual attention network in children with attention deficit/hyperactivity disorder (ADHD). Frontiers in Human Neuroscience, 8, A 0162 10.3389/fnhum.2014.00162. PMC3960496 24688465

[ref64] Zhang, S. , McIntosh, J. , Shadli, S. M. , Neo, P. S. H. , Huang, Z. , & McNaughton, N. (2017). Removing eye blink artefacts from EEG—A single-channel physiology-based method. Journal of Neuroscience Methods, 291, 213–220. 10.1016/j.jneumeth.2017.08.031. 28860078

